# Comparison of three commercial AI tools for detection and malignancy assessment of incidental lung nodules

**DOI:** 10.3389/fmed.2026.1806511

**Published:** 2026-06-03

**Authors:** Oguzhan Tokur, Oyunbileg von Stackelberg, Luca Dulz, Bettina K. Budai, Manuel Debic, Nan Wei, Claus P. Heussel, Hans-Ulrich Kauczor, Mark O. Wielpütz

**Affiliations:** 1 Department of Diagnostic and Interventional Radiology, Heidelberg University Hospital, Heidelberg, Germany; 2 Translational Lung Research Center Heidelberg (TLRC), German Center for Lung Research (DZL), Heidelberg, Germany; 3 Department of Diagnostic and Interventional Radiology with Nuclear Medicine, Thoraxklinik at University of Heidelberg, Heidelberg, Germany; 4 Department of Radiology, Kütahya Health Sciences University, Kütahya, Türkiye; 5 Department of Diagnostic Radiology and Neuroradiology, University Medicine Greifswald, Greifswald, Germany; 6 Department of Nuclear Medicine, University Medicine Greifswald, Greifswald, Germany

**Keywords:** artificial intelligence (AI), cancer, computed tomgraphy (CT), lung, nodule

## Abstract

**Objectives:**

Incidental pulmonary nodules are common on computed tomography (CT), and management typically relies on size and volume. Artificial intelligence (AI)-based tools show promise in nodule detection and risk assessment, but their clinical utility remains uncertain. This study aimed to evaluate the accuracy of AI-based software in detecting and predicting the malignancy of incidental pulmonary nodules.

**Materials and methods:**

This retrospective study included patients selected from a cohort of 1,138 individuals who underwent chest CT between 2015 and 2024 and met the inclusion criteria. Patients were classified into benign and malignant groups. Malignancy was determined by histopathology or by at least 2 years of follow-up. Nodule location, size, and type were assessed using by both using radiology reports and AI tools. Three commercial tools (AI-I, AI-II, and AI-III) were assessed for nodule detection and malignancy risk prediction. Agreement was assessed using Cohen’s kappa and the intraclass correlation coefficient (ICC), and diagnostic performance was evaluated using receiver operating characteristic analysis.

**Results:**

Chest CT scans of 374 patients (mean age 66 ± 9 yr.; range 37–88 yr.; 231 males) with at least one solid or part-solid nodule were evaluated. AI-I and AI-II demonstrated excellent agreement with radiology reports for nodule localization (*κ* = 0.95, *p* < 0.001) and moderate agreement for nodule type (*κ* = 0.46, *p* < 0.001). Size assessment showed excellent agreement with ICC values of 0.93 [95%CI = 0.92–0.94] for AI-I and 0.89 [95%CI = 0.86–0.91] for AI-II. AI-II differed from AI-III in malignancy prediction with AUC = 0.77 [95%CI = 0.72–0.81] and 0.89 [95%CI = 0.85–0.92], respectively. Additionally, AI-II showed significantly lower PPV (65.57% vs. 87.20%, *p* < 0.001) and accuracy (72.1% vs. 82%, *p* < 0.001) than AI-III.

**Conclusion:**

AI-based tools demonstrated high accuracy for incidental pulmonary detection; however, their performance in malignancy risk stratification differed substantially.

## Introduction

Incidental pulmonary nodules are common findings in radiological practice, requiring management based on their morphology and clinical characteristics (1, 2). Most nodules are benign, and identification of malignancy is crucial for early diagnosis and potentially curative treatment. On the other hand, aggressive strategies can lead to unnecessary invasive diagnostic and therapeutic procedures, increasing morbidity, mortality, and healthcare costs (3, 4). Accurate detection and risk stratification of incidental pulmonary nodules constitute a substantial part of the workload of chest radiologists.

In routine clinical practice, guidelines are utilized to determine whether further evaluation is required for incidental nodules, based on morphological characteristics and clinical information (2, 5, 6). Despite the widespread use of these guidelines, increasing hospital workloads and the difficulty in accessing each patient’s clinical information can lead to errors in nodule detection and assessment (7, 8). Although certain quantitative methods have been utilized to improve diagnostic accuracy, the development of numerous artificial intelligence (AI)-based tools, designed to assist in the detection and management of pulmonary nodules, has increased, and studies have demonstrated that such tools can also support less experienced radiologist by enhancing their diagnostic performance (9–11). AI software approved by the U.S. Food and Drug Administration (FDA) for clinical use has primarily been developed for nodule detection, with some also capable of malignancy risk prediction (12). Currently, commercially available AI-based software is mainly trained using screening CT datasets, but can also be applied to incidental nodules despite the divergent background risk. Since each software utilizes different algorithms, datasets for training, reference standards, or evaluation metrics, comparisons between them are challenging. We hypothesized that employing different AI-based software tools for nodule detection and risk prediction may lead to different clinical management decisions. Accordingly, we compared two commercially available AI-based software tools for the detection of incidental pulmonary nodules. Furthermore, we compared the accuracy of malignancy prediction of another set of two software tools in nodules that were pathologically confirmed or had a definite outcome after at least 2 years of follow-up. The primary endpoints were the diagnostic accuracy of these tools in nodule detection and malign risk assessment, while secondary endpoints included nodule measurements, morphology, and anatomical distribution.

## Materials and methods

### Cohort and image acquisition

The study was approved by the local ethics committee (S-005/2017) and conducted in accordance with the principles of the Declaration of Helsinki. The requirement for informed consent was waived.

Patients aged ≥35 who underwent chest CT (Somatom Definition AS 64/Eco, Somatom Volume Zoom, or Somatom Emotion 6, Siemens Healthineers, Forchheim, Germany) at our institution between 2015 and 2024 with solid and part-solid incidental nodules were retrospectively included. CT scans were reconstructed with 1.5 mm slice-thickness (1.0 mm increment) with soft- (b40f, i40f) and lung kernels (b70f, i70f). Images with motion artifacts were excluded. 107 examinations were performed with i.v. contrast material (Imeron 300, Bracco, Italy). For these, the mean density in Hounsfield Units (HU) was measured with an ROI placed in the aortic arch. In line with the recommendation of one manufacturer (Optellum, Oxford, UK), those reaching >300HU were excluded.

**Figure 1:**
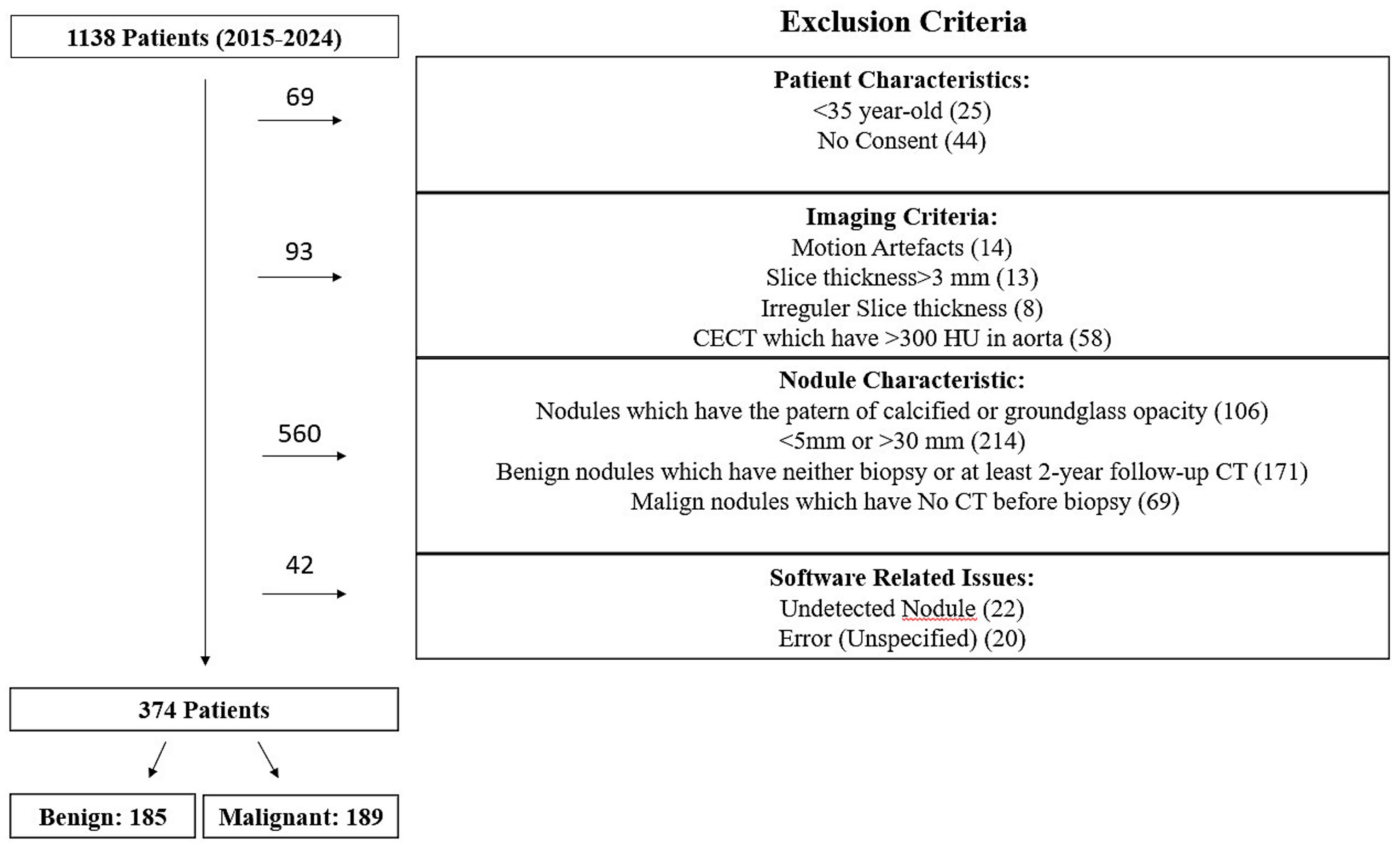
Study flowchart.

The most suspicious or largest single nodule of each patient was evaluated. To ensure comparability, exclusion criteria for nodule characteristics that were compatible with all AI software were applied. Accordingly, nodules <5 mm or >30 mm were excluded. Further, due to the exclusion criteria of one of the manufacturers (Optellum, Oxford, UK), pure ground-glass nodules (GGO) and calcified nodules were excluded from the cohort. Datasets that could not be evaluated by any of the software were excluded. A total of 374 patients with at least one nodule constituted the final study group (Figure 1), comprising 143 (38.2%) females and 231 (61.8%) males. The mean age was 66 years [range 37–88]. There was no statistically significant difference in age between the benign and malignant groups (*p* = 0.81) (Table 1).

**Table 1:** Patient and nodule characteristics.

Characteristics	Categories	Benign	Malignant	Total	*p*
		*n* (%)	*n* (%)	*n* (%)	
SEX					0.78
	Male	113 (61.1)	118 (62.4)	231 (61.8)	
	Female	72 (38.9)	71 (37.6)	143 (38.2)	
Location					<0.05
	RUL	48 (25.9)	60 (31.7)	108 (28.9)	
	RML	20 (10.8)	12 (6.3)	32 (8.6)	
	RLL	44 (23.8)	43 (22.8)	87 (23.3)	
	LUL	23 (12.4)	43 (22.8)	66 (17.6)	
	LLL	50 (27.1)	31 (16.4)	81 (21.7)	
Type					0.45
	Solid	179 (96.8)	180 (95.2)	359 (96)	
	Part-solid	6 (3.2)	9 (4.8)	15 (4)	
Method					<0.05
	Follow-up	145 (78.4)	0 (0)	145 (38.8)	
	Pathology	40 (21.6)	189 (100)	229 (61.2)	
Image					<0.05
	Contrast (+)	38 (20.5)	96 (50.8)	131 (35.0)	
	Contrast (−)	147 (79.5)	93 (49.2)	243 (65)	
Total		185 (49.5)	189 (50.5)	374 (100)	
Size (mm)	Mean± SD (min-max)	9 ± 3 (5–25)	15 ± 5 (6–29)	12 ± 5 (5–29.3)	<0.05
Age (YEARS)	Mean± SD (min-max)	66 ± 10 (40–88)	66 ± 9 (37–84)	66 ± 9 (37–88)	0.79

RUL, Right Upper Lobe; RML, Right Middle Lobe; RLL, Right Lower Lobe; LUL, Left Upper Lobe; LLL, Left Lower Lobe; SD, Standard Deviation; min: minimum; max, maximum; P, *p*-value.

### Image evaluation

Three commercially available AI-based software tools were evaluated independently and compared with one another: Siemens AI-Rad Companion Chest CT (version LungCAD VD20, Siemens Healthineers, Erlangen, Germany) (AI-I), Aview LCS (version v1.1.46.11-win, Coreline Soft., Seoul, Korea) (AI-II), and Virtual Nodule Clinic (Optellum, Oxford, UK) (AI-III). The outputs provided by AI-based software tools are summarized in Table 2.

**Table 2:** Endpoints generated by the three different artificial intelligence (AI)-based software tools.

Characteristics	Report	AI-I	AI-II	AI-III
Detection	X	X	X	
Measurements (diameter, volume)	X	X	X	
Type (solid, part-solid, ground glass)	X	X	X	
Localization (lobe)	X	X	X	
Lung-RADS	X		X	
Malignancy score				X

Respective variables for comparison in two separate analyses are highlighted in grey. Radiology reports served as the standard of reference for detection and measurements, whereas histology and/or long-term follow-up served as the standard of reference for diagnosis of malignancy.

The size, type, and localization of the related nodules were obtained from the radiology reports of the patients, and the reports generated by the AI-based software were compared by an expert radiologist. For assessing the diagnostic accuracy in distinguishing malignant from benign nodules, pathology results (biopsy and/or surgery) or at least 2 years of follow-up imaging were considered as the standard of reference. The last CT scan before histological diagnosis was analyzed with each AI-based software. Nodules with at least 2 years of follow-up imaging, which did not show changes suggestive of malignancy during this period according to Fleischner criteria, were considered benign (2).

**Figure 2:**
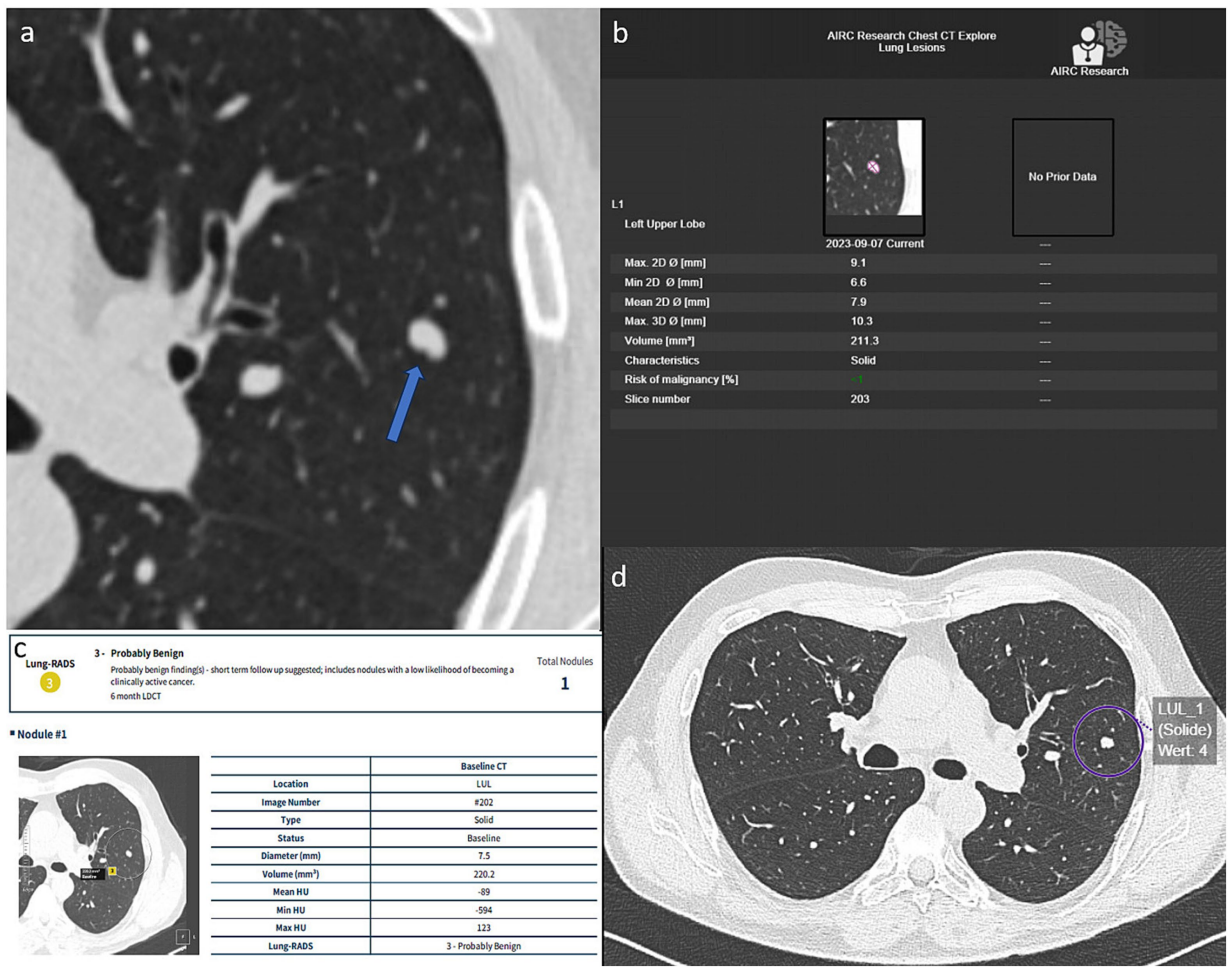
Results of a benign nodule in AI-based software tools: **(a)** Magnified axial image of the nodule (arrow), **(b)** AI-I report of the nodule, **(c)** AI-II report, **(d)** AI-III risk assessment.

**Figure 3:**
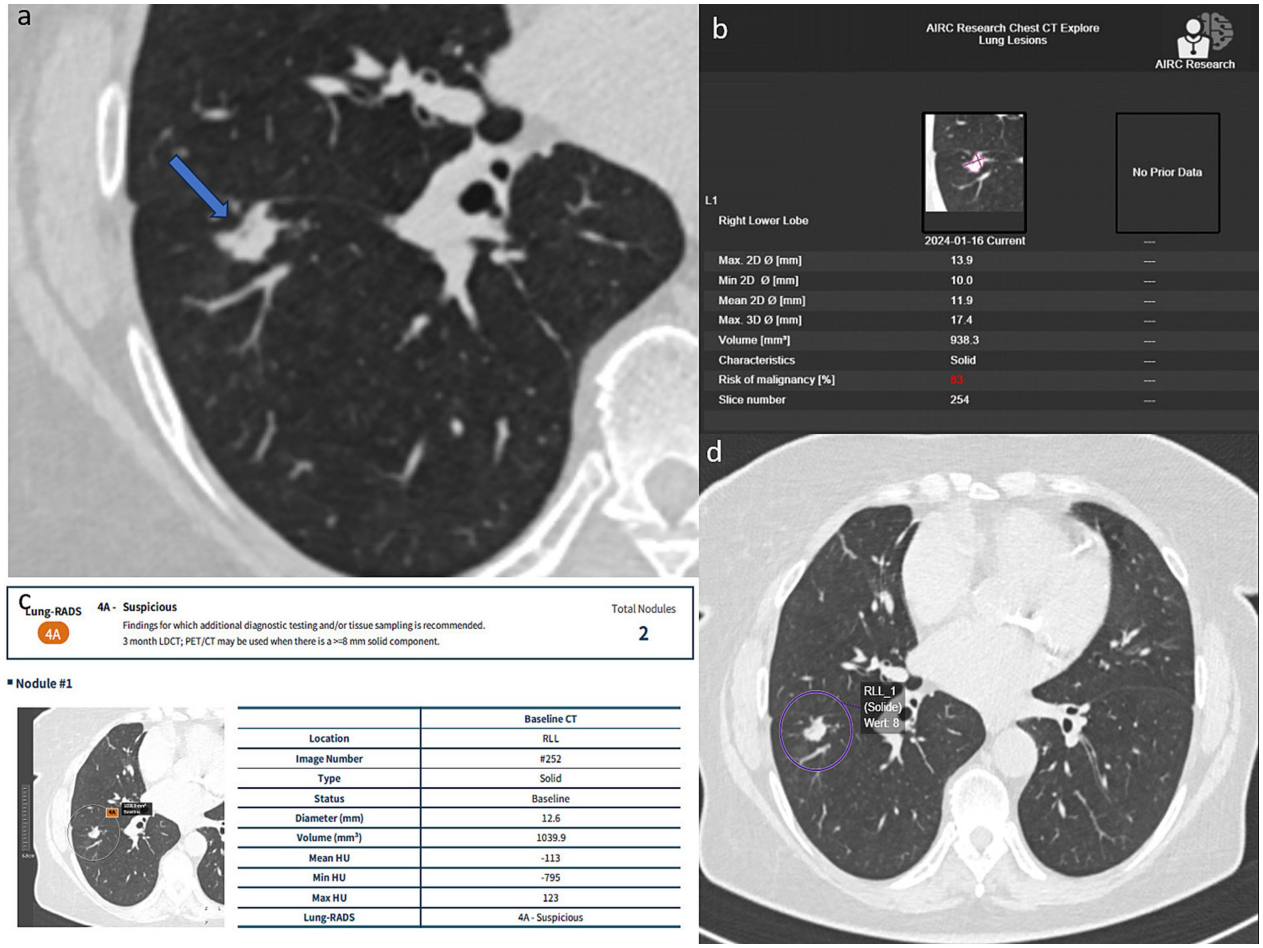
Results of a malignant nodule in AI-based software tools: **(a)** Magnified axial image of the nodule (arrow), **(b)** AI-I report of the nodule, **(c)** AI-II report of the nodule, **(d)** AI-III risk assessment.

The three AI tools differed in the respective outputs, allowing for direct comparison through clearly prespecified primary and secondary endpoints (Table 2). Our primary endpoints were the diagnostic accuracy of the tools in nodule detection (comparing AI-I vs. AI-II) and their performance in malignancy risk prediction (comparing AI-II vs. AI-III) (Table 3). Our secondary endpoints included the evaluation of nodule measurements (diameter and volume), morphological type, and anatomical localization (comparing AI-I vs. AI-II), using the original radiology reports as the standard of reference (Table 4). The Lung-RADS category of each patient was retrospectively assigned based on the type and size of the nodule in the original radiology reports, and was subsequently included in the comparative analysis. AI-II generated Lung-RADS categories, whereas AI-III provided a malignancy score reflecting the estimated clinical risk of malignancy. Cut-offs of Lung-RADS >4A (AI-II) and malignancy score of >7 (AI-III) were set as thresholds for indicating high risk of malignancy, allowing for statistical comparison (10, 13, 14). The Lung-RADS categories generated by AI-II and the malignancy scores provided by AI-III are CE-certified and approved for clinical use (see Figures 2, 3).

**Table 3:** Performance criteria of AI-based software tools in lung nodule assessment.

Diagnostic performance metrics	REPORT	AI-II	AI-III
True positive *n* (%)	180 (48.1)	179 (47.9)	143 (38.2)
True negative *n* (%)	76 (20.3)	91 (24.3)	164 (43.9)
False positive *n* (%)	109 (29.1)	94 (25.1)	21 (5.6)
False negative *n* (%)	9 (2.4)	10 (2.7)	46 (12.3)
Total *n* (%)	374 (100)	374 (100)	374 (100)
Sensitivity (95% CI)	95.24 (91.15–97.8)	94.71 (90.49–97.43)	75.66 (68.9–81.6)
Specificity (95% CI)	41.08 (33.92–48.54)	49.19 (41.78–56.63)	88.65 (83.17–81.6)
PLR (95% CI)	1.62 (1.43–1.83)	1.86 (1.61–2.16)	6.67 (4.42–10.05)
NLR (95% CI)	0.12 (0.06–0.22)	0.11 (0.06–0.20)	0.27 (0.21–0.35)
PPV (95% CI)	62.28 (59.32–65.16)	65.57 (62.21–68.78)	87.20 (81.87–91.13)
NPV (95% CI)	89.41 (81.35–94.24)	90.10 (83.03–94.42)	78.10 (73.39–82.17)
Accuracy (95% CI)	68.45 (63.47–73.13)	72.19 (67.35–76.68)	82.09 (77.82–85.84)
AUC (95% CI)	0.76 (0.72–0.81)	0.77 (0.72–0.81)	0.89 (0.85–0.92)

PLR, Positive Likelihood Ratio; NLR, Negative Likelihood Ratio; PPV, Positive Predictive Value; NPV, Negative Predictive Value; AUC, Area Under the Curve; CI, Confidence Interval. Cut-off values of Lung-RADS > 4A and malignancy score > 7 were set for comparability.

**Table 4:** Nodule characteristics in AI-based software tools and radiology reports.

Characteristics	Categories	Benign	Report	Total	AI-I			AI-II		
			Malignant		Benign	Malignant	Total***	Benign	Malignant	Total***
		*n* (%)	*n* (%)	*n* (%)	*n* (%)	*n* (%)	*n* (%)	*n* (%)	*n* (%)	*n* (%)
Location^*^	RUL	48 (25.9)	60 (31.7)	108 (28.9)	47 (25.4)	57 (30.2)	104 (27.8)	50 (27)	61 (32.3)	111 (29.7)
	RML	20 (10.8)	12 (6.3)	32 (8.6)	23 (12.4)	10 (5.3)	33 (8.8)	16 (8.6)	9 (4.8)	25 (6.7)
	RLL	44 (23.8)	43 (22.8)	87 (23.3)	42 (22.7)	47 (24.9)	89 (23.8)	46 (24.9)	44 (23.3)	90 (24.1)
	LUL	23 (12.4)	43 (22.8)	66 (17.6)	23 (12.4)	42 (22.2)	65 (17.4)	24 (13)	44 (23.3)	68 (18.2)
	LLL	50 (27.1)	31 (16.4)	81 (21.7)	50 (27)	33(17.5)	83 (22.2)	49 (26.5)	31 (16.4)	80 (21.3)
Type^*^	Solid	179 (96.8)	180 (95.2)	359 (96)	183 (98.9)	181 (95.8)	364 (97.3)	164 (88.9)	178 (94.2)	342 (91.4)
	Semisolid	6 (3.2)	9 (4.8)	15 (4)	1 (0.5)	8 (4.2)	9 (2.4)	16 (8.6)	10 (5.3)	26 (7)
	Groundglass	0 (0)	0 (0)	0 (0)	1 (0.5)	0 (0)	1 (0.3)	5 (2.7)	1 (0.5)	6 (1.6)
Total		185 (49.5)	189 (50.5)	374 (100)	185 (100)	189 (100)	374 (100)	185 (100)	189 (100)	374 (100)
Size^**^ (mm)	Mean ± SD (min-max)	9 ± 3 (5–25)	15 ± 5 (6–29)	12 ± 5 (5–29.3)	10 ± 3 (4–24)	16 ± 5 (6–37)	13 ± 5 (4–37)	9 ± 3 (3–27)	13 ± 4 (5–27)	11 ± 4 (3–27)
Volume^**^ (mm^3^)	Mean ± SD (min-max)				465 ± 884 (15–9,634)	1,502 ± 1,456 (16–6,637)	989 ± 1,313 (15–9,634)	635 ± 1,036 (21–10,433)	1828 ± 1856 (99–10,848)	1,238 ± 1,619 (21–10,848)

RUL, Right Upper Lobe; RML, Right Middle Lobe; RLL, Right Lower Lobe; LUL, Left Upper Lobe; LLL, Left Lower Lobe; SD, Standard deviation; min: minimum; max, maximum; *Cohen’s kappa value; **Intraclass correlation coefficient; ***p < 0.001.

### Statistical analyses

All statistical analyses were conducted using SPSS (version 25.0, IBM Corp., Armonk, NY, USA, 2017), MedCalc software (version 20.115; Ostrend, Belgium), RStudio (version 2026.01.2) and R software. The Shapiro–Wilk test was performed to assess normality. Continuous variables are presented as mean ± standard deviation, and categorical variables as percentages. Student’s *t*-test and Mann–Whitney U test were applied for continuous variables, and the chi-square test for categorical variables. Cohen’s kappa and intraclass correlation coefficient (ICC) were used to assess agreement between AI-based software and radiology reports. Area under the curve (AUC) in a receiver operating characteristic (ROC) analysis was used to indicate the diagnostic performance of AI-based software. Accuracy, sensitivity, specificity, positive predictive value (PPV), and negative predictive value (NPV) were computed using Youden’s index.

To evaluate the clinical utility of the AI tools beyond standard diagnostic accuracy, Decision Curve Analysis (DCA) was performed to calculate the net benefit across a range of threshold probabilities. To assess the reliability of the malignancy risk predictions, calibration curves were plotted and Brier scores were calculated for both AI tools. Additionally, to evaluate the robustness of the algorithms across different imaging protocols, subgroup analyses were conducted to compare the diagnostic performance between contrast-enhanced and non-contrast CT scans. A *p* < 0.05 was considered statistically significant.

## Results

### Benign and malignant nodules show different localization and properties

Malignant nodules were more prevalent in the upper lobes (*p* = 0.004), and were larger in size (*p* < 0.001). Ground-glass nodules were not included in the cohort; no statistically significant difference in nodule type was observed between the groups (*p* > 0.05). There were no significant differences between the groups regarding clinical factors such as sex and age (Table 1).

### AI-based tools automatically detect and measure dimensions of incidental pulmonary nodules

AI-I and AI-II agreed almost perfectly with radiology reports and with each other regarding nodule localization (*κ* = 0.95 for both, *p* < 0.001). The agreement between AI-I and AI-II, and radiology reports for nodule type was moderate (*κ* = 0.44, 0.46, 0.46, respectively; *p* < 0.001).

For the most suspicious nodule, the detection sensitivity was 93.8% for AI-I and 96.2% for AI-II (Table 2). Both software tools failed to detect a total of six nodules: four nodules located on the diaphragmatic surface and two nodules adjacent to the mediastinum.

AI-I and AI-II demonstrated excellent agreement with radiology reports for nodule dimension measurements [ICC = 0.93 (95% CI = 0.92–0.94) and 0.89 (95% CI = 0.86–0.91), respectively]. In the study group, the smallest and largest dimensions in AI-I were 4 mm (15 mm^3^) and 37 mm (9.6 cm^3^), respectively, whereas in AI-II, they were 3 mm (21 mm^3^) and 27 mm (10.8 cm^3^). The agreement between the tools for nodule size was excellent [ICC = 0.92 (95% CI = 0.91–0.94) (Table 4)].

### AI-based tools differ in malignancy risk prediction for incidental pulmonary nodules

We further compared the risk stratification performance of Lung-RADS categories derived from radiology reports and AI-II, as well as the malignancy scores generated by AI-III, against the reference standard of benign versus malignant nodule classification. The AUC for predicting malignancy was 0.76 (95% CI = 0.72–0.81, *p* < 0.001) for radiology reports, 0.77 (95% CI = 0.72–0.81, *p* < 0.001) for AI-II, and 0.89 (95% CI = 0.85–0.92, *p* < 0.001) for AI-III. The accuracy of radiology reports (68%) and AI-II (72.1%) was similar, whereas AI-III reached superior results (82%) (Table 3). Additionally, decision curve analysis showed that the AI-III generally provided the highest net benefit across most clinically relevant threshold probabilities, consistently outperforming the AI-II, and radiologist-based models. At a threshold probability of 0.20, the net benefit values were 0.424 for AI-III, 0.408 for the radiologist-based model, and 0.396 for AI-II. At a threshold of 0.30, AI-III remained superior (0.392), followed by AI-II (0.371), the radiologist-based model (0.356). At a threshold of 0.50, the separation became more pronounced, with net benefit values of 0.321 for AI-III, 0.227 for AI-II, and 0.190 for the radiologist-based model. At a higher threshold of 0.70, the net benefit of the radiologist-based model declined further to 0.117, indicating reduced clinical utility at stricter decision thresholds. Overall, the preservation of the general ranking of models across a broad range of threshold probabilities supports the robustness of the decision-analytic findings to reasonable variations in threshold selection. When the sensitivity analysis was restricted solely to the pathologically confirmed subgroup, AI-III maintained significantly superior diagnostic performance over AI-II (*p* < 0.001), despite a decrease in their respective AUC values (from 0.89 to 0.74, and from 0.77 to 0.59).

Calibration plot assessment showed that the bootstrap bias-corrected calibration curve closely approximated the ideal reference line, indicating good agreement between predicted probabilities and observed outcomes. The mean absolute error was 0.022. In addition, Brier score analysis supported the superior overall probabilistic performance of the AI-III, which yielded the lowest Brier score (0.130), followed by AI-II (0.184), and the radiologist-based model (0.185). These findings indicate better overall probabilistic accuracy for the AI-III, with comparatively lower performance for the other approaches. Calibration intercepts were close to 0 and calibration slopes were 1.00 for all models in the apparent-performance analysis; however, these estimates were derived from the same dataset used to fit the logistic recalibration models and should therefore be interpreted as apparent rather than optimism-corrected calibration metrics.

In subgroup analyses according to contrast administration, the overall ranking of models remained broadly consistent. In the contrast-enhanced subgroup, the AI-III provided the highest net benefit across most clinically relevant threshold probabilities, whereas AI-II showed intermediate performance and the radiologist-based model demonstrated comparatively lower clinical utility. A similar pattern was observed in the non-contrast subgroup, in which the AI-III again yielded the greatest net benefit across most relevant thresholds, while the remaining model showed lower net benefit overall. These findings support the robustness of the main decision-analytic results across contrast-enhanced and non-contrast CT examinations.

## Discussion

In this study, we evaluated the performance of three commercially available AI-based software tools, originally developed primarily for the assessment of nodules on screening chest CT, in a real-world clinical setting involving a broader spectrum of patient groups and various chest CT examinations with incidentally detected nodules. In particular, we focused on their performance in characterizing the most suspicious nodule and in predicting malignancy risk. Accordingly, both AI-I and AI-II showed excellent agreement with radiology reports in terms of nodule localization and size. However, we observed a considerably lower agreement in determining the nodule type, which we believe is due to the lack of adequate part-solid nodules in the study. When comparing the malignancy predictions of AI-III and AI-II against histology results or at least 2 years of follow-up, we obtained better results with AI-III. AI-III performs risk stratification for nodules based on a specifically trained neural network, while AI-II uses Lung-RADS based on measured nodule properties, which provides management categories rather than a true risk estimation for cancer.

One of the important factors in the risk stratification of nodules is their type. Guidelines provide different management approaches based on the type of nodule (15, 16). Many FDA-approved AI-based software tools for nodule detection also provide information on nodule size, localization, and type. Ciompi et al. (17) developed an algorithm capable of determining nodule type in six categories, demonstrating its reliability in categorizing pulmonary nodules. In our study, AI-I showed good agreement with radiology reports in nodule type determination, while AI-II showed moderate agreement. However, the evaluation in our study is limited by the lack of ground-glass opacity nodules due to the inclusion criteria and by the relatively low number of part-solid nodules compared to solid nodules.

Among the guidelines for nodule management, Lung-RADS is one of the most widely used. According to the literature, the malignancy prediction accuracy of Lung-RADS scoring can reach up to 0.8 in ROC analysis (18). Similarly, in our study, the AI-II using Lung-RADS for risk assessment achieved an AUC of 0.77. Higher values of 0.89 were observed with AI-III. The primary distinction lies in the fact that Lung-RADS provides a categorical approach for nodule management, whereas AI-III offers a continuous risk stratification system for nodule evaluation (15). In addition, while Lung-RADS is primarily designed for screening purposes, the risk stratification system employed by AI-III has been developed to encompass a broader range of clinical applications (10, 13). Therefore, head-to-head statistical comparisons of diagnostic accuracy between these two different systems cannot be perfectly balanced or completely fair. In the study by Mikhael et al. (19), they developed a model that achieved high accuracy, with an area under the curve (AUC) ranging from 0.86 to 0.94, for predicting the risk of malignancy. Another study conducted by Ardila et al. (20) demonstrated that their algorithm (AUC: 0.92) achieved higher accuracy than radiologists in cancer risk assessment. Unlike our study, their work focused on screening populations in the risk group. In our study, although the AI-based software tools were trained on mainly screening populations, we evaluated their performance on a more diverse range of patient groups across various chest CTs in daily practice with incidental nodules. This broader application may explain the lower success in malignancy prediction.

From a clinical decision-making perspective, these findings indicate that the AI-III is more likely to provide the greatest practical benefit across a broad range of malignancy threshold probabilities. In other words, when clinicians vary in how aggressively they classify pulmonary nodules as malignant, the AI-III appears to better preserve the balance between identifying true-positive cases and avoiding unnecessary interventions. AI-I also demonstrated meaningful clinical utility, although its net benefit remained lower than that of AI-III across most clinically relevant threshold ranges. In contrast, the AI-II and radiologist-based models showed a more limited utility profile, with a clearer decline at moderate-to-high thresholds, suggesting less robust performance when stricter malignancy thresholds are applied.

Peters et al. (21) evaluated the performance of the LCP-CNN algorithm, which utilizes a 1–10 malignancy risk scoring system, and reported a sensitivity of 58.2% in identifying malignant pulmonary nodules as high risk (score ≥ 9) under standard-dose CT conditions. Their study also demonstrated that virtual CT dose reduction applied after image acquisition may impact the algorithm’s performance, potentially leading to changes in malignancy risk classification. In our study, a similar scoring-based AI model achieved a higher sensitivity of 75.66%. This discrepancy may be attributed to differences in cohort characteristics and cut-off value selection.

Although employing different commercial AI models and methodology, a recent study by Herber et al. (22) evaluated 158 pathologically confirmed pulmonary nodules and reported AUC values between 0.57 and 0.63 for malignancy risk assessment. In contrast, our study demonstrated higher diagnostic performance, with AUC values ranging from 0.77 to 0.89. This discrepancy may stem from methodological differences, including the variance in cohort sizes. Furthermore, the inclusion of follow-up-proven stable nodules as benign cases in our study—whereas Herber et al. (22) relied exclusively on histopathological confirmation from a potentially higher-risk, biopsied or resected cohort—could be a primary reason for this observed difference in AI performance.

This study has limitations. First, the strict exclusion criteria, mandated by the technical limitations of the software (excluding pure GGOs, calcified nodules, and nodules <5 mm or >30 mm), resulted in a highly selective cohort with a nearly 1:1 distribution of benign to malignant nodules. This enriched case-mix does not represent the typical incidental nodule prevalence in the general population, which inherently distorts predictive values (such as PPV and NPV) and may reduce the generalizability of our findings to the broader incidental nodule population. Moreover, using original radiology reports as the reference standard for nodule detection and measurement is highly practical but methodologically weaker than a rigorous, independent expert panel re-review. Second, the comparability of the risk score with Lung-RADS risk categories is limited. While the risk score has been validated both in the setting of lung cancer screening and incidental nodules, Lung-RADS is validated only in the setting of lung cancer screening, which cannot readily be transferred. Additionally, AI-II and AI-III used different algorithms and classification schemes (Lung-RADS vs. malignancy score); moreover, contrast-enhanced chest CTs were also included in the study, which may further limit direct comparability. Yet, other validated classification systems, which could be used for the purpose of our study, are missing. Third, while adhering to other inclusion criteria in our study, criteria requiring access to clinical information were not utilized, as obtaining such data is not always feasible in daily routine. Fourth, the limited number of part-solid nodules resulted in an insufficient sample for detection and risk assessment. Furthermore, analyzing only the single most suspicious or largest nodule per patient does not fully reflect routine clinical practice, where the presence of multiple nodules frequently influences patient management. Last, as the inclusion criteria for benign nodules were not restricted to histopathological confirmation, slowly growing, subtle cancers may be missed by the follow-up regime.

In conclusion, characterization and malignancy prediction of incidental pulmonary nodules are essential for early diagnosis and treatment, as well as for avoiding unnecessary interventions and treatments caused by false positives. AI-based software tools showed high accuracy in detection, but differences in malignancy risk prediction may stem from their diverse structural outputs (categorical vs. continuous risk scores) rather than strict clinical superiority.

## Data Availability

The datasets presented in this article are not readily available because data could be available after the request from corresponding author with an convenient explanation. Requests to access the datasets should be directed to oguzhantokur@gmail.com.
